# Commentary: Systems Biology Approach to Model the Life Cycle of *Trypanosoma cruzi*

**DOI:** 10.3389/fcimb.2017.00001

**Published:** 2017-01-18

**Authors:** Alejandra Carrea, Luis Diambra

**Affiliations:** ^1^Laboratorio de Biología de Sistemas, Centro Regional de Estudios Genómicos, Facultad de Ciencias Exactas, Universidad Nacional de La PlataLa Plata, Argentina; ^2^Consejo Nacional de Investigaciones Científicas y TécnicasArgentina

**Keywords:** gene regulatory network, *Trypanosoma cruzi*, metabolic enzymes, moonlighting proteins, computational approaches

In a recent work we have identified, from a bigger gene regulatory network, a seven-node module involved in the control of the life cycle of *Trypanosoma cruzi* (*T. cruzi*) (Carrea and Diambra, 2016). To that end, we have analyzed microarray gene-expression data of the four different *T. cruzi*'s life cycle stages, by means of a systems biology approach. The found module is the smallest gene regulatory subnetwork able to emulate the dynamical properties of the parasite. This module is composed of nine genes: three of them coding for uncharacterized proteins, and the other six genes coding for characterized proteins. The latter code for: a hexokinase, a δ-1-pyrroline-5-carboxylate dehydrogenase, a quinone oxidoreductase, a glutamate dehydrogenase, a peptidyl-prolyl *cis-trans* isomerase, and a metaciclina II. Except for metaciclina II, these genes code for proteins involved in metabolic pathways. Thus, we were expecting gene-expression regulatory proteins instead of the striking information we obtained. Yet, it eventually became clear that these metabolic enzymes could have other regulatory functions beyond their known metabolic one. This type of multifunctional proteins are known as moonlighting proteins (Jeffery, 1999). They were first discovered in the late 1980s by Piatigorsky et al. (1988). They found that the lens structural protein δ-crystallin and the metabolic enzyme argininosuccinate lyase are both encoded by the same gene in ducks. Today, it is well-known that moonlighting proteins comprise diverse kinds of proteins, and that they are present in many different organisms including animals, plants, yeasts, prokaryotes, and protists (for reviews see Jeffery, 2009; Huberts and van der Klei, 2010; Jeffery, 2014).

Moonlighting proteins characterized so far in unicellular parasites are mostly enzymes. Examples of these parasite's moonlighting enzymes include: a hexokinase in *Leishmania donovani*, which functions as a hemoglobin receptor within the parasite's flagellar pocket; the glycolytic enzyme aldolase in *Toxoplasma gondii* and *Plasmodium falciparum*, which has an additional (even if non-essential) function in host-cell invasion; various soluble metabolic enzymes in *Trichomonas vaginalis*, which moonlight as adhesins allowing parasite-host cell adhesion; the mitochondrial peroxiredoxin in *Leishmania infantum*, which not only functions as a peroxidase, but also as a chaperone essential for pathogenesis; the α-ketoglutarate dehydrogenase E2 in bloodstream *Trypanosoma brucei*, which moonlights in the mitochondria ensuring a correct kinetoplast DNA inheritance (Collingridge et al., 2010; Ginger, 2014). Recently, Ferreira and colleagues have shown that the enzyme mevalonate kinase, originally involved in sterol isoprenoids biosynthesis in *T. cruzi*'s glycosomes, is also secreted and may modulate host cell signaling during parasite invasion (Ferreira et al., 2016).

A search carried out in two different moonlighting protein databases (MoonProt: http://www.moonlightingproteins.org Mani et al., 2015, and MultitaskProtDB: http://wallace.uab.es/multitask Hernández et al., 2014) showed that for the five metabolic enzymes that comprise the above mentioned module, there exist homologous enzymes in other organism/s with proven moonlighting function/s (Table 1). Another fact that came to light from this search is that, until now, there is no record of proteins moonlighting in *T. cruzi* in these two databases. Furthermore, there are several other examples in literature showing that some of these five enzymes actually moonlight, or have the potential to do so, in different organisms. We have previously mentioned hexokinase moonlighting as a hemoglobin receptor in *Leishmania donovani* (Collingridge et al., 2010). Mantilla and colleagues have proposed that *T. cruzi*'s δ-1-pyrroline-5-carboxylate dehydrogenase has additional functions beyond the production of glutamate (Mantilla et al., 2015). They have suggested that this enzyme could be involved in the infection process, interacting with components of the mammalian host cells. They have based this hypothesis in enzyme structural data, in its mitochondrial membrane localization, and in its higher activity observed during the infective stages of the parasite. Another example is the peptidyl-prolyl *cis-trans* isomerase known as Mip, which moonlights as a host collagen IV biding protein in *Legionella pneumophila*. Rasch and colleagues have identified a 13-aminoacid-long peptide in collagen IV as the target of Mip, and have found that blocking this binding causes a decrease in bacterial transmigration *in vitro* (Rasch et al., 2014).

**Table 1:** **Moonlighting functions of the five enzymes that comprise the module controlling the *T. cruzi*'s life cycle in other organisms, according to MoonProt and MultitaskProtDB**.

**Enzyme Name**	**Uniprot ID of the Homologous Enzyme in *T. cruzi***	**Moonlighting Function (Organism)**	**Reference**
Hexokinase	Q4D3P5	Glucose signaling - Porine binding - Apoptosis - Intracellular glucose sensor (*A. thaliana*)	Moore et al., 2003
		Apoptosis (*E. coli*)	Sukumaran et al., 2010
		Transcriptional regulation (*S. cerevisiae*)	Moreno and Herrero, 2002
δ-1-pyrroline-5-carboxylate dehydrogenase	Q4DRT8	Transcriptional repression of the put operon (*S. typhimurium*)	De Spicer et al., 1991
			De Spicer and Maloy, 1993
		Transcriptional repression of the put operon (*E. coli*)	Wood, 1981
Quinone oxidoreductase	Q4DHH8	Lens crystallin (*C. porcellus*)	Rao et al., 1992
		Lens crystallin (*H. japonica*)	Fujii et al., 2001
Glutamate dehydrogenase	Q4DWV8	Transcription factor binding activity (*B. subtilis*)	Gunka et al., 2010
Peptidyl-prolyl *cis-trans* isomerase	Q4E4L9	Induces apoptosis of gastric epithelial cells - Activates monocyte IL-6 synthesis (*H. pylori*)	Basak et al., 2005
		Extracellular function: proinflammatory cytokine (*H. sapiens*)	Jin et al., 2004

Taking all of the above into consideration, it follows that not limiting the functions of a given protein to its canonical one, while keeping in mind that it could be a moonlighting protein, is of the uppermost importance. Moreover, in the search of new therapeutic targets, it would be interesting and useful to investigate these moonlighting functions in previously well characterized proteins. In doing so, it must be remembered that moonlighting proteins constitute a major challenge when it comes to predicting their functions based solely on sequence homology or on conserved domains. This analysis could lead to the loss of some proteins' functions or to the assignment of wrong functions to other proteins (Jeffery, 2014, 2015). To overcome this difficulty, computational approaches combining several types of data (available omics-scale data, functional annotations in public databases, bioinformatics predictions, computational simulations of pathways, molecular dynamics of biomolecules, and other biochemical data) have been developed (Khan and Kihara, 2014; Khan et al., 2014). These integrative computational strategies will allow us to deepen our knowledge of moonlighting proteins and, therefore improve the design of drugs that must affect only the desired function of the target protein (Khan and Kihara, 2014), and will also help us understand how and why these proteins can be essential during infection, virulence or immune responses (Jeffery, 2014, 2015).

Finally, in relation to *T. cruzi*'s pathogenesis, all these progresses could increase our current knowledge of host-parasite interactions, and help accelerate the discovery of effective drugs against Chagas disease. In a systems biology context, moonlighting proteins can function as a link or switch between two different pathways, and aid the cell to respond to environmental changes (Jeffery, 2014, 2015). Transitions between two different developmental stages in *T. cruzi* may be triggered by environmental changes such as the availability of nutrients or energy sources. To mention one representative example, it was demonstrated that L-proline amino acid plays an essential role during intracellular differentiation of *T. cruzi* in the mammalian host (Tonelli et al., 2004). This raises the possibility that enzymes participating in metabolic pathways could be, in addition, moonlighting proteins. Therefore, we suggest that the five metabolic enzymes resultant from our previous work (Carrea and Diambra, 2016) should be considered when studying *T. cruzi* and its implications in human health.

## Author contributions

AC and LD conceived and designed the work, and analyzed and interpreted the data. AC wrote the manuscript. LD critically revised the manuscript. AC and LD approved the final manuscript.

## Funding

AC is a postdoctoral fellow of the CONICET (Argentina). LD is a research member of the CONICET (Argentina).

### Conflict of interest statement

The authors declare that the research was conducted in the absence of any commercial or financial relationships that could be construed as a potential conflict of interest.
